# Supplementary material for: Integrative analysis of gene expression and alternative splicing in microalgae grown under heterotrophic condition
Source: PLoS One. 2020 Jun 17;15(6):e0234710. doi: 10.1371/journal.pone.0234710 (PMC7299362; doi:10.1371/journal.pone.0234710)
Supplement: Attachment — Submitted filename: article_comments.pdf [file pone.0234710.s005.pdf]

The article is a well-written, needed, and helpful to understand the relationship between growth conditions and differential gene expression pattern. The interesting thing that I liked is how alternative splicing events can change the mode of metabolism in response to growth condition change. I thought this article and previous article about identification of functional modules and regulatory hubs may help us to study the principal factors that can change specific metabolism to get specific product for commercial production.

1. Kindly correct the word “evenets”. (Abstract-line No.5)
