# Supplementary material for: Integrative analysis of gene expression and alternative splicing in microalgae grown under heterotrophic condition
Source: PLoS One. 2020 Jun 17;15(6):e0234710. doi: 10.1371/journal.pone.0234710 (PMC7299362; doi:10.1371/journal.pone.0234710)
Supplement: Attachment — Submitted filename: Response to Reviewers.docx [file pone.0234710.s006.docx]

Reviewer comments

Response to Editor comments

Dear editor thank you for your useful comments.

we revised the manuscript thoroughly revised. As your comment we separated results and dissuasion sections and revised these sections. Regarding the validation with wet lab experiments, unfortunately *Auxenochlorella protothecoides* is not available for our institution and also contrary because of limitation of biological material transformation. Therefore, we had to validate the results with another RNA seq data sets follow the procedure that has been proposed by Feng et al. 2018. Characterization of kinase gene expression and splicing profile in prostate cancer with RNA-Seq data. BMC Genomics.

Regarding the differences with our previous published manuscript, in our previous manuscript, we focused on connectivity analysis and functional module detection in network analysis, where as in current study we analysis gene expression and alternative splicing with comparative approach.

Reviewer #1:

Kindly correct the word “evenets”. ( Abstract-line No.5)

Response: It was revised

Reviewer #2:

This work appears to bring new knowledge on the transcriptional and post-transcriptional responses of alga towards the growth conditions influenced by nutrient inputs.

However, the details of nutrient parameters in two conditions are not stated by the authors and also how they are influencing above process.

It was revised

It should require more clarifications for claiming their suitability to novelty.

General Editing comments:
The paper requires a detailed round of editing. There are some examples of missing legends and references in the text. The user should be able to read the caption and have all the information required to understand the figure or table.

It was revised

Specific Comments:

a) Add a brief description of the autotrophic and heterotrophic culture conditions and nutrient parameters used in this study. Because this work emphasizes the growth conditions and it will help in understanding the context.

It was revised

b) Mention the correct test and control sample size and their replications; ie. In the material and methods section Page No. 9 first paragraphs (The sample size of autotrophic growth conditions and their test timings).

It was revised

c)Page number 11, figure 2B: The data presented here are quite interesting. However, authors need to make a connection between these data with the possible importance/influence of the algal metabolite production.

Dear reviewer in the end of the “discussion” section we discussed this relationships

d) In the result section, functional impact of DEG and DAS genes, in third paragraph. The authors state that “Transcription regulator activities of DEGs are significant …” Are these statistical differences observed after the analytical experiment? If so, where are the stats to prove the statements in this paragraph? If a statistical analysis was completed, please add the test statistic and p values either embedded in the text or in a graph figure legend.

Dear reviewer enrichment analysis thoroughly tested based on statically methods implemented in WEGO. we included (P-Value<0.05) to show significance of enriched ontology in the text and figure legend.

e)Figure legend ‘C’ is present in the text, but absent in all mentioned figures.

It was revised

f)Page No. 8 reference ‘Huang & Xu, 2015’ is present in the text, but absent in the reference list

It was revised

g)Page No. 15 line number 13th journal description is present in the reference section but author detail is absent in text as well as in reference.

It was revised

h)Page No. 15 Kong et al. Journal page number missing

It was revised

i)Page No. 15 Panahi et al., typographic error in year.

It was revised

j)Page No. 16 Ravichandran et al. and Xiao et al. References journal page number missing.

It was revised

k)Page No. 16 Panahi et al., typographic error in journal page number

It was revised

l)Page No. 16 Schurch & Schofield (2016) Journal name not mentioned

It was revised

m)Reference journal name consistency; either follow full name/abbreviation/italic pattern/non italic.

Dear reviewer it was revised
